# Feeding the invaders: how metabolic imbalance shapes infection and biofilm development

**DOI:** 10.1093/femsre/fuag029

**Published:** 2026-06-30

**Authors:** Sahil Mahajan, Sarah MacDowell, Cortney Mitchem, Evan Alexander, Daniel J Wozniak

**Affiliations:** Department of Microbial Infection and Immunity, The Ohio State University, Columbus, OH 43210, United States; Division of Pulmonary, Critical Care, and Sleep Medicine, Department of Internal Medicine, College of Medicine, The Ohio State University, Columbus, OH 43210, United States; Department of Microbial Infection and Immunity, The Ohio State University, Columbus, OH 43210, United States; Department of Microbiology, The Ohio State University, Columbus, OH 43210, United States; Department of Microbial Infection and Immunity, The Ohio State University, Columbus, OH 43210, United States; Department of Microbial Infection and Immunity, The Ohio State University, Columbus, OH 43210, United States; Department of Microbiology, The Ohio State University, Columbus, OH 43210, United States

**Keywords:** metabolic diseases, immune dysfunction, bacterial persistence, inflammation, biofilms

## Abstract

Infectious and metabolic disorders have risen sharply and pose a significant threat to human populations worldwide. Despite being deemed unrelated, mounting evidence indicates that metabolic disorders markedly heightened both the likelihood and severity of infectious disease. The mechanism underlying this association is only beginning to be appreciated and involves a complex interaction between altered metabolic state, pathogen virulence mechanisms, and immune effector pathways. Here, we highlight that a dysregulated metabolic environment disrupts homeostasis, creating a nutrient-rich, immunocompromised milieu that promotes bacterial survival and replication in the host. Metabolic dysfunction is also discussed as a factor that promotes bacterial persistence by altering reactive oxygen species production and inducing tissue hypoxia, conditions that favor biofilm growth. We also examine how bacteria directly exploit nutrient surplus in metabolic diseases to boost their virulence programs and biofilm formation. Moreover, given the ever-increasing problem of antibiotic resistance, we discuss how metabolic diseases contribute to this challenge. Collectively, uncovering how metabolic disorders increase chronic or recurrent infections can guide the development of effective treatment strategies.

## Teaser

People with metabolic disorders, such as obesity, diabetes, and dyslipidemia (panel A) are at higher risk of severe bacterial infections. Elevated nutrient levels in these individuals directly promote bacterial growth, compromise the immune system, and increase the susceptibility to biofilm formation and the development of antibiotic resistance (panels B and C).

## Introduction

Metabolic diseases have become a significant global health crisis, affecting individuals across all ages, genders, and geographies. Conditions such as hyperglycemia, hypertension, dyslipidemia, obesity, and diabetes have been progressively rising because of a physically passive lifestyle, intake of highly processed foods that are high in calories and lack dietary fiber, socioeconomic factors, and inherited risk due to genetic susceptibility (Hotamisligil 2006, Grundy 2008, Delpino et al. 2022, Chew et al. 2023, Suzuki et al. 2024, Zhang et al. 2024, Noubiap et al. 2025). According to the World Health Organization, ~2.5 billion adults globally are overweight. Roughly 890 million of them are classified in the category of clinically obese (Phelps et al. 2024). Of particular concern is the significant rise in obesity rates among children, as 35 million children under the age of 5 are overweight (Edition of the Joint Child Malnutrition Estimates 2025). Just as alarming, an estimated 390 million children and adolescents aged 5–19 years are overweight. Of these, about 160 million are living with obesity (Ge et al. 2025). With obesity becoming increasingly prevalent, the number of people living with diabetes has increased by more than four-fold in just over 20 years (Zhou et al. 2024). This increased trend is also reflected in the rising prevalence and mounting mortality rates associated with individuals who have dyslipidemia (Wang et al. 2023, Trimarco et al. 2024, Ballena-Caicedo et al. 2025). Beyond these health challenges, the long-term economic impact of metabolic diseases is also rising sharply (Okunogbe et al. 2022, Chong et al. 2024). Yet, the pace of progress in preventing and treating these diseases remains inadequate, leaving a significant gap in addressing the clinical needs of those affected.

Research across various metabolic diseases consistently demonstrates that an imbalance in the composition of metabolites and their utilization within individual cells, specific organs, and systemically plays a key role in the onset of these disorders. Indeed, people with metabolic disorders exhibit significant alterations in the major classes of metabolites, including carbohydrates, amino acids, and lipids (Wylie-Rosett et al. 2004, Elshorbagy et al. 2012, Klop et al. 2013, Takashina et al. 2016, Petrus et al. 2020, Alshuweishi et al. 2024). Under steady state, the human body drives for energy efficiency and favors the storage of excess calories. However, when this is coupled with a caloric surplus and excessive nutrient intake, the additional calories ultimately lead to increased adiposity and related metabolic conditions (Smith et al. 2018, Blüher 2019). In fact, metabolic disorders greatly increase the risk of cardiovascular and neurodegenerative diseases (Blüher 2019, Capucho et al. 2022, Maack 2025), multiple types of cancers (Vigneri et al. 2009, Lauby-Secretan et al. 2016), as well as an increased susceptibility and severity of infections (Falagas and Kompoti 2006, Twig et al. 2018, Darwitz et al. 2024, Carey et al. 2025a, Nyberg et al. 2026). The increased risk of infection in metabolic diseases results from several factors that affect both the host and the microbe. These factors include elevated nutrient levels that favor pathogen growth, weakened immune responses, and an ongoing chronic low-grade inflammation (Collins et al. 2024, Darwitz et al. 2024, Hu et al. 2024). Metabolic disorders also influence the response to antibiotic therapy, as exemplified by the rise in antibiotic resistance among individuals suffering from diabetes. This increased burden of antimicrobial resistance in people with metabolic diseases is likely multifactorial, arising from the combined effects of increased antibiotic use and metabolic disease-related biological changes (Venmans et al. 2009, Shook et al. 2025). These disorders are also suggested to be linked with inadequate vaccine responses (Young et al. 2013, Park et al. 2014, Geerling et al. 2022). Strikingly, both the risk and severity of infections are consistently higher for individuals suffering from obesity, diabetes, and dyslipidemia (Falagas and Kompoti 2006, Darwitz et al. 2024, Holt et al. 2024, Benn et al. 2025, Nyberg et al. 2026). This highlights the need to explore how metabolic disturbances in these disorders increase the risk of infection and influences disease outcomes.

Upon infection, immune cells detect the microbes and microbial products and change the way they produce and use the energy, a process known as immunometabolism. This metabolic switch allows immune cells to rapidly produce energy, supporting their proliferation and enhancing their effector functions (Mathis and Shoelson 2011, O’Neill et al. 2016). However, metabolic disorders can disrupt these pathways, weakening the immune system and compromising its ability to eliminate pathogens. The disruption in pathogen clearance, coupled with continuous microbial exposure, leads to low-grade chronic inflammation. The sustained inflammation keeps immune cells continuously activated, ultimately leading to immune exhaustion and lowering their capacity to mount robust, pathogen-specific responses (Erlandsson et al. 2024, Braga Tibaes et al. 2025, Jee et al. 2025). Over time, this constant activation creates metabolic stress and leads to the accumulation of metabolic waste products, including reactive oxygen species (ROS) and inflammatory metabolites. This disrupts immune cell energy use and reinforces a chronic inflammatory state (Lasselin and Capuron 2014, Furman et al. 2019). On the other hand, empirical evidence shows that chronic inflammation contributes to the onset of metabolic diseases and plays a significant role in advancing their progression (Hotamisligil 2006). This interdependent relationship between metabolic diseases and infections creates a vicious cycle in which each condition worsens the other, leading to persistent infections and progressive metabolic decline. At the core of this interplay lies immune cell metabolic rewiring, which represents a central pathogenic mechanism driving chronic inflammation, immune dysfunction, and persistent infection in metabolic disorders.

In healthy individuals, an infected tissue often becomes a nutrient-restricted environment where the host and pathogen compete for essential resources. The host actively employs mechanisms that limit the pathogen access to nutrients while ensuring that sufficient metabolic fuel is preserved for tissue function and for mounting an effective immune response (Berney and Berney-Meyer 2017, Kaiser and Heinrichs 2018, Kedia-Mehta and Finlay 2019, Horn and Kielian 2021, Chen et al. 2025). However, metabolic diseases disrupt this balance, creating a nutrient-rich tissue environment that favors bacterial colonization and growth (Irmal et al. 1999, Falagas and Kompoti 2006, Twig et al. 2018, Nyberg et al. 2026). Research has shown that higher level of nutrients, such as glucose, are linked with increased bacterial virulence mechanisms. These mechanisms, including increased tissue adherence and biofilm formation, contribute to the chronicity and persistence of infection (Vitko et al. 2016, Butrico et al. 2023, O’Connor et al. 2024). Increased glucose also impairs multiple arms of the immune system, thereby diminishing the hosts ability to clear early infections before biofilms are established (Table 1). Therefore, metabolic disorder-associated metabolic changes alter infection outcomes and represent an underappreciated area in infection biology.

**Table 1:** Metabolic disease-associated factors that can influence biofilm formation.

Metabolic diseases-associated factor	Effect on biofilm formation	Suggested mechanism
ROS imbalance and oxidative stress	Promotes biofilm initiation and development	• Oxidative stress triggers genetic mutations that elevate c-di-GMP levels, directly driving the evolution of biofilm-forming bacterial variants in *Pseudomonas aeruginosa* (Chua et al. 2016). • Oxidative stress regulates bacterial motility and attachment, thereby initiating biofilm formation (Strempel et al. 2017). • ROS drive mucoid conversion in *P. aeruginosa* by inducing hyperproduction of the exopolysaccharide alginate (Mathee et al. 1999, Ramsey and Wozniak 2005, Limoli et al. 2014).
Hypoxia and anoxia	Promotes biofilm formation and maturation	• Anoxia increases expression of the exopolysaccharide PIA that stabilizes *Staphylococcus aureus* biofilms (Cramton et al. 2001). • Hypoxia promotes increased bacterial cell lysis, leading to the release of eDNA, which reinforces the biofilm matrix (Mashruwala et al. 2017). • Hypoxia drives mucoid conversion in *P. aeruginosa* by inducing hyperproduction of the exopolysaccharide alginate (Worlitzsch et al. 2002).
Chronic hyperglycemia	Promotes biofilm formation	• Excess glucose induces EPS production (Seidl et al. 2008, She et al. 2019) • Glucose promotes biofilm formation by lowering intracellular c-di-AMP levels (Syed et al. 2024).
Advanced glycation end products (AGE)	Promotes biofilm formation	• AGE enhance biofilm formation by promoting eDNA release (Xie et al. 2020).
Impaired immune responses	Promotes biofilm formation	• Weakens initial bacterial control (Darwitz et al. 2024).

The rising incidence of antibiotic treatment failure in those with metabolic disorders, partly due to greater bacterial resistance to antimicrobial compounds, underscores the urgent need to understand the metabolic interactions between infiltrating immune cells and bacteria in metabolically dysregulated tissues. This review aims to focus on the following fundamental questions: (1) How do bacterial infections alter immunometabolism, and how do bacteria exploit these pathways for their benefit? (2) How metabolic diseases reshape immunometabolism and susceptibility to infection. (3) How do metabolic disorders alter bacterial metabolism and virulence pathways? (4) How do metabolic derangements affect the efficacy of antimicrobial therapies?

### Immunometabolism at the host–pathogen interface

The immune system’s ability to fight infections and maintain homeostasis is closely dependent on underlying metabolic activity. Metabolism–immune interaction has emerged as an important area of study, revealing how metabolic pathway profoundly shapes immune cell function. Metabolic pathways in immune cells directly determine their fate and function by regulating energy supply and biosynthetic pathways required for activation, differentiation, and effector functions (Pearce 2010, Simsek et al. 2010, Kelly and Aj O’neill 2015). These pathways are highly flexible and can change in response to distinct immune phenotypes, a process known as metabolic reprogramming. This adaptability enables immune cells to efficiently utilize available resources, especially under nutrient-limited conditions (Kelly and Aj O’neill 2015, Netea et al. 2016, Mills et al. 2017). During infection, this reprogramming acts as an adaptive response, significantly altering carbohydrate, amino acid, and lipid metabolism to meet increased energy and biosynthetic demands of activated immune cells (Michalek et al. 2011, Pearce and Pearce 2013, Everts et al. 2014, Nakaya et al. 2014, O’Sullivan et al. 2014, Yan and Horng 2020). Understanding how each nutrient class contributes to energy production, biosynthesis, and cellular signaling is essential for understanding the regulation of immune responses under both normal conditions and during infection. In this section, we will examine how carbohydrates, amino acids, and lipids influence immune cell function to support effective host defense. We will also discuss how bacteria can take advantage of these metabolic changes to enhance their survival.

### Carbohydrate metabolism during infection

**Figure 1:**
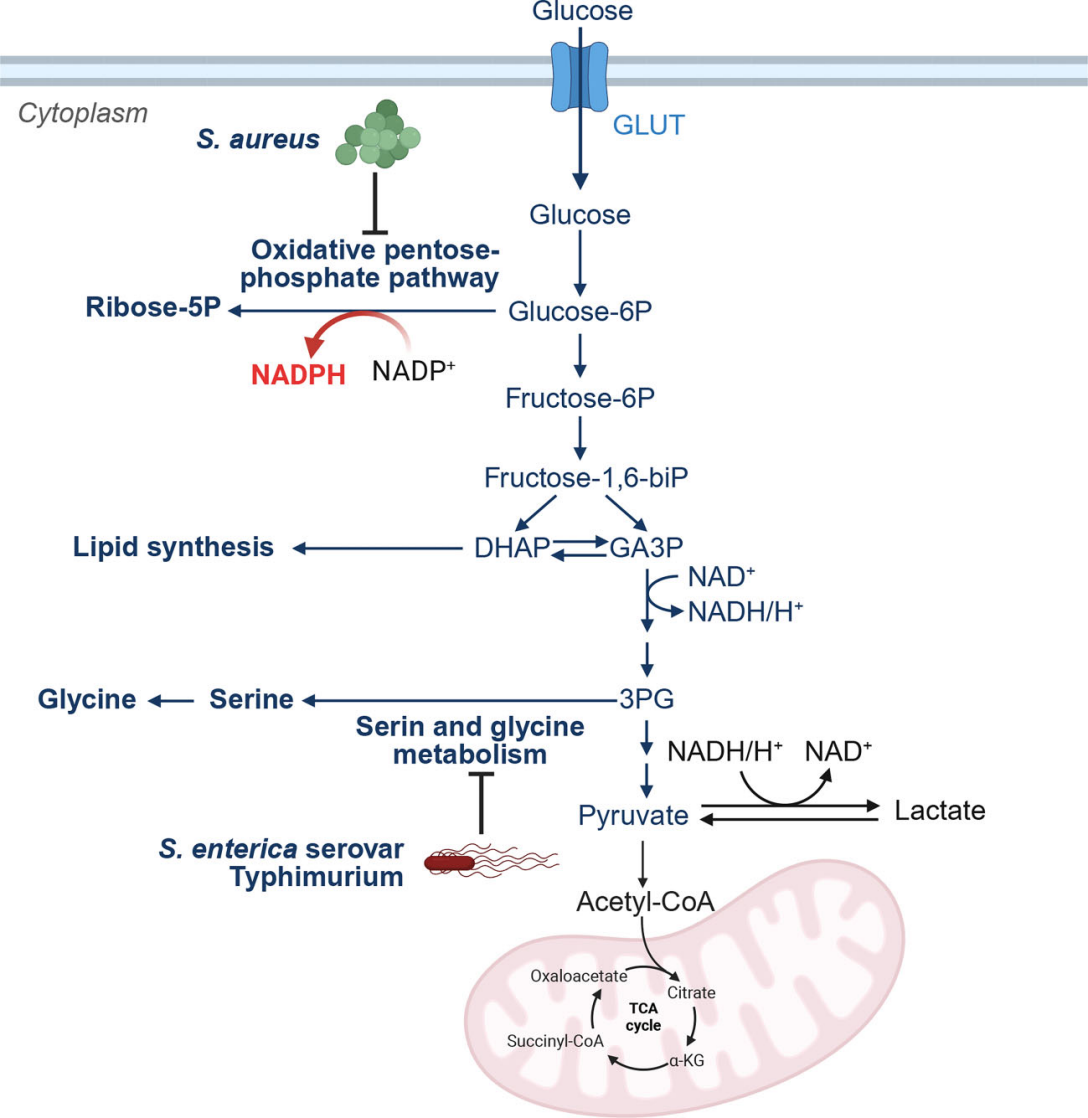
Schematic representation of the fate of glucose upon infection. Warburg-like metabolic changes involve the conversion of glucose to pyruvate followed by its reduction to lactate, despite an intact and fully functional respiratory change. Infection also leads to the accumulation of glycolytic intermediates, which increases the precursors for biosynthetic pathways required for immune cell activation. The increased levels of glucose-6-phosphate (Glucose-6P) promote the flux through the oxidative PPP, DHAP is diverted towards lipid synthesis, while 3-phosphoglycerate (3PG) supports serine and glycine metabolism. Pathogens can exploit these biosynthetic pathways to support their own survival and replication within the host. *Staphylococcus aureus* attenuates the oxidative PPP and *S. enterica* serovar Typhimurium decreases serine synthesis, thereby modulating the host metabolic processes to their advantage. Created in https://BioRender.com.

Under steady-state, immune cells remain largely quiescent and rely primarily on oxidative phosphorylation (OXPHOS) to meet their metabolic needs (Fox et al. 2005, Jones and Thompson 2007, Wculek et al. 2023). In this metabolic environment, functional mitochondria fully oxidize glucose in the presence of oxygen, yielding CO_2_ and water. This process represents the most efficient way of generating ATP to support the basic cellular functions of resting immune cells. However, upon infection, immune cells quickly multiply to fight the invading pathogens. The expansion of immune cells is an energy-intensive process that requires increased metabolic activity. To meet these heightened energy demands, immune cells switch to glycolysis even when oxygen is available, a phenomenon known as the Warburg effect (Borregaard and Herlin 1982, Fox et al. 2005, Jones and Thompson 2007, Heiden et al. 2009, Krawczyk et al. 2010) (Fig. 1). This metabolic rewiring allows immune cells to rapidly generate ATP while funneling glycolytic intermediates into pathways required for proliferation and effector function. For example, during infection, glucose-6-phosphate derived from glycolysis is shunted into the oxidative pentose phosphate pathway (PPP) to generate ribose-5-phosphate and NADPH (Nagy and Haschemi 2015, Britt et al. 2022, TeSlaa et al. 2023). The ribose-5-phosphate serves as a critical precursor for nucleotide synthesis, while NADPH helps maintain the redox balance. The resulting increase in nucleotide biosynthesis is essential for the rapid proliferation of immune cells. Glucose-6-phosphate dehydrogenase (G6PD), the rate-limiting enzyme of PPP, plays a critical role in facilitating this process. Importantly, *Staphylococcus aureus* suppresses the PPP in human neutrophils, thereby enhancing bacteria intracellular survival (Vozza et al. 2024) (Fig. 1). In agreement with this, disruption of PPP by loss of G6PD impairs immune cell functions and markedly increases infection susceptibility (Spolarics et al. 2001, Sun et al. 2022, Alrahmany et al. 2023). In parallel, glycolytic intermediates also serve as the precursors for amino acid synthesis. For example, 3-phosphoglycerate, another intermediate of glycolysis, contributes to the biosynthesis of serine and glycine (Amelio et al. 2014, He et al. 2023). These amino acids, in turn, support the production of cytokines, chemokines, antibodies, complement proteins, antimicrobial peptides, and other effector molecules necessary for mounting an effective immune response (Kelly and Pearce 2020, Ling et al. 2023). Pathogens can undermine this metabolic dependency, as illustrated by *Salmonella enterica* serovar Typhimurium, which uses the effector protein SopE2, delivered through the type III secretion system (T3SS), to suppress host serine synthesis (Fig. 1). The reduction results in the accumulation of 3-phosphoglycerate, which the bacteria utilize as a nutrient source to support intracellular growth (Jiang et al. 2021). Finally, glycolytic intermediates are also involved in lipid biosynthesis. For example, dihydroxyacetone phosphate (DHAP) is reduced by glycerol-3-phosphate dehydrogenase to glycerol-3-phosphate, which serves as the backbone of membrane phospholipid and is essential for cellular membranes (Agranoff and Hajra 1971, Cristobal et al. 2024) (Fig. 1). These lipids also promote granule formation and trafficking, facilitating the release of antimicrobial peptides and proteases that provide critical defense against microbial infection (Meer Van et al. 2008, Pol van der et al. 2012). Certain bacteria, such as *Rickettsia prowazekii*, import host DHAP to generate glycerol-3-phosphate using its own dehydrogenase, hijacking this pathway for their benefit (Frohlich et al. 2010). Overall, glycolysis serves as the central driver of immune metabolism during infection.

### Amino acid metabolism during infection

Infection also reshapes protein and amino acid metabolism to support the acute phase response. Immune cells respond to pathogen invasion by tightly regulating amino acid availability, increasing their catabolism, and producing metabolites with antimicrobial and immunomodulatory functions (Grohmann and Bronte 2010, Mcgaha et al. 2012, Tomé 2021). A well-studied example is arginine metabolism, which hinges on the coordinated activity of opposing enzymatic pathways, namely, nitric oxide synthases (NOS1, NOS2, and NOS3) and arginases (Arg1 and Arg2) (Morris 2007, Fung et al. 2025). All NOS isoforms catalyze the breakdown of l-arginine into nitric oxide (NO) and l-citrulline (Andrew and Mayer 1999). While NOS1 and NOS3 are constitutively expressed and produce low levels of NO primarily for signaling, NOS2 is strongly induced by microbial and inflammatory stimuli in macrophages and produces high levels of NO that are critical for effective pathogen killing (MacMicking et al. 1997, Förstermann and Sessa 2011). On the other hand, arginase is an alternative pathway for arginine metabolism, catalysing the hydrolysis of l-arginine to l-ornithine and urea (Caldwell et al. 2018, Li et al. 2022a). The arginase pathway supports downstream polyamine and collagen biosynthesis, which are important for wound healing and tissue repair (Wynn 2004). Arginase isoforms exhibit distinct expression patterns, cellular localizations, and induction triggers across the immune cell landscape. Arg1 is expressed in macrophages, myeloid-derived suppressor cells, and dendritic cells, and is stored in neutrophil granules where it is released upon degranulation (Mantovani et al. 2009, Rotondo et al. 2011a, Rodriguez et al. 2017). Its expression is induced by Th2 cytokines, such as IL-4, IL-13, and IL-10, as well as M-CSF and certain pathogen-associated stimuli (Munder et al. 1998, Pauleau et al. 2004). In contrast, Arg2 is not broadly expressed in many immune cells and is mainly induced under stress conditions (Suwanpradid et al. 2014). Interestingly, the balance between NOS2 and Arg1 expression in macrophages determines their functional polarization. Classically activated macrophages increase NOS2 expression to produce high levels of NO to fight pathogens, while Arg1 expression remains low (MacMicking et al. 1997, McWhorter et al. 2013). In contrast, alternatively activated macrophages mainly express Arg1, promoting tissue repair and suppressing NOS2 activity and T cell responses (Wynn 2004, Li et al. 2022b). Several pathogens capitalize on this metabolic switch to evade immune clearance. For example, *Mycobacterium tuberculosis* induces Arg1 expression, which depletes l-arginine (Qualls et al. 2010). This limits the availability of l-arginine for NOS2-dependent NO production. Meanwhile, *Helicobacter pylori* promote Arg2 expression in macrophages, thereby lowering NOS2 activity and limiting bacterial killing (Lewis et al. 2010). Infection also reprograms tryptophan metabolism by activating indoleamine 2,3 dioxygenase (IDO1) (Schmidt and Schultze 2014). IDO1 breaks down tryptophan into kynurenine, depleting local tryptophan levels in infected tissue (Seo and Kwon 2023). The tryptophan depletion starves effector T cells, triggering GCN2-dependent anergy and apoptosis that impair their function (Fallarino et al. 2002, Munn et al. 2005). Meanwhile, kynurenine acts as a ligand for the aryl hydrocarbon receptor (Opitz et al. 2011), which promotes regulatory T cell differentiation and IL-10 production. Together, these mechanisms suppress adaptive immunity. The tryptophan depletion also affects tryptophan-auxotrophic bacteria, such as *Chlamydia trachomatis, Coxiella burnetii*, and group B Streptococcus, during infection. This starves the bacteria, preventing their replication and enhancing immune clearance. Some bacteria, such as *M. tuberculosis*, leverage host tryptophan starvation by synthesizing their own tryptophan to establish chronic infection. Another example of amino acid reprogramming during infection is the alteration of glutamine metabolism. Immune cells, such as macrophages, increase glutamine uptake to meet increased energy demands. When glucose becomes limited during infection, glutamine supports the tricarboxylic acid cycle (TCA) through glutaminolysis. In glutaminolysis, glutamine is first converted into glutamate by glutaminase. Glutamate is subsequently transformed to α-ketoglutarate, a TCA cycle intermediate, by glutamate dehydrogenase. Beyond supporting the TCA cycle, glutamine donates nitrogen atoms for purine and pyrimidine biosynthesis, thereby supporting immune cell proliferation. Some intracellular pathogens, like *Chlamydia* species, consume all host-derived glutamine for peptidoglycan synthesis, which is essential for their replication. Other bacteria, such as *Listeria monocytogenes* and *Yersinia enterocolitica*, detect high glutamine levels within macrophages to activate their virulence factors (Lee et al. 2001, Haber et al. 2017). These factors then block phagolysosome formation and prevent further phagocytosis by other immune cells. These bacteria also induce macrophage cell death, ensuring their extracellular survival and replication. In summary, amino acid metabolism restricts nutrients for pathogens, but in some cases, they capitalize on it to aid their survival.

### Lipid metabolism during infections

Infection also rapidly reprograms lipid metabolism. At steady-state, dietary fats are absorbed in the intestine and packaged into chylomicrons. These chylomicrons transport triglycerides, cholesterol, and fat-soluble vitamins from the gut into the bloodstream. Lipoprotein lipase, anchored on capillary walls, hydrolyzes triglycerides in chylomicrons, releasing free fatty acids (FFA) for uptake and use by peripheral tissues. Once most triglycerides are removed, the liver clears the remaining chylomicron remnants through apolipoprotein E-mediated receptor uptake. The liver then processes the unutilized lipids and repackages them into very low-density lipoprotein for delivery to peripheral tissues (Mansbach and Siddiqi 2010, Dash et al. 2015). Under homeostatic conditions, immune cells primarily depend on circulating lipids and basal fatty acid oxidation to meet their modest energy needs and limited requirement for membrane biogenesis (Li et al. 2026). During infection, however, immune cells undergo extensive metabolic reprogramming, characterized by increased lipid uptake mediated by scavenger receptors (SRs), like CD36 and SR-A1, and fatty acid-binding proteins (Prado et al. 2023). In parallel, the immune cells also activate intrinsic de novo lipogenesis (DNL), a process in which carbohydrates are converted into fatty acids and lipids to support their cellular functions (Kidani et al. 2013, Berod et al. 2014). These anabolic lipid programs are tightly regulated at the transcriptional level by sterol regulatory element-binding proteins (SREBPs), which control the expression of genes involved in lipid synthesis and uptake (Eberlé et al. 2004, Jeon and Osborne 2012, Ricoult et al. 2015). The newly synthesized and acquired lipids are subsequently stored in lipid droplets, which function as a reservoir of neutral lipids. Beyond storage, these lipid droplets act as sinks for excess fatty acids, preventing lipotoxic stress (Olzmann and Carvalho 2018, Prevost et al. 2025). They also provide lipids to support membrane expansion, organelle biogenesis in proliferating immune cells, and the production of lipid-mediated inflammatory mediators (Bozza and Viola 2010, Blot et al. 2025). Interestingly, bacteria can exploit host lipid metabolic pathways to acquire nutrients, build their own membranes, and create niches that support their survival and replication. For example, *M. tuberculosis* as an intracellular pathogen exploits host-derived lipids, including cholesterol and fatty acids, as its carbon and energy source to persist within macrophages (Pandey and Sassetti 2008, Lee et al. 2013). *Mycobacterium tuberculosis* use specialized transporters, such as Mec4 and Mec1for cholesterol and fatty acid uptake, respectively, to acquire these lipid nutrients (Pandey and Sassetti 2008, Nazarova et al. 2017). In addition, during infection, host lipid droplets interact with mycobacteria-containing phagosomes, blocking their maturation and subsequent fusion with lysosomes, thereby ensuring bacterial survival (Melo and Dvorak 2012, Roque et al. 2020). Similarly, *Legionella pneumophila* activates SREBP1 to hijack host DNL, supplying lipids that support the expansion of legionella-containing vacuole within the host cell (Abshire et al. 2016, Ondari et al. 2022). Altogether, lipid metabolism serves as a critical interface between host defense and pathogen survival.

### Inflammation and immune dysfunction in metabolic disorders

The link between metabolism and inflammation depends on a tightly regulated balance, normally maintained through short-term compensatory and adaptive mechanisms. When this coordination is disrupted, and either metabolic or inflammatory signaling dominates for too long, the resulting imbalance can lead to detrimental physiological outcomes. Chronic activation of immune cells disrupts their normal metabolism, while long-term metabolic problems can, in turn, trigger aberrant immune responses. This dysregulation is a defining feature of metabolic disorders, which are characterized by sustained immune cell activation and elevated levels of proinflammatory cytokines (Saltiel and Olefsky 2017). Clinically, this state is highlighted by increased circulating levels of cytokines, such as TNFα and IL-6, high-sensitivity C-reactive proteins, and fibrinogen (Malenica et al. 2017, Stanimirovic et al. 2022). Importantly, this low-grade inflammation develops in the absence of any overt infection. It is referred to as sterile inflammation and stems primarily from metabolic stress rather than pathogen-derived signals. Overtime, persistent low-grade inflammation in metabolic diseases constantly put pressure on the immune system, leading to tissue damage and exhaustion of the immune system (Donath and Shoelson 2011, Huang et al. 2024).

One of the key mechanisms linking metabolic dysfunction to chronic inflammation is insulin signaling. Beyond its classical role in glucose regulation, insulin functions at the interface between metabolism and immunity, influencing nutrient balance (Schenk et al. 2008), immune cell activation (Helderman and Strom 1978, Makhijani et al. 2023), and inflammatory responses (Luca de and Olefsky 2007). Secreted by pancreatic β cells, insulin is essential in maintaining glucose and lipid homeostasis. Its signaling is initiated when insulin binds to its receptor on the surface of target cells, leading to receptor autophosphorylation and subsequent recruitment and phosphorylation of insulin receptor substrates (IRS). Phosphorylated IRS activates PI3K-Akt signaling, promoting glucose uptake by enhancing the trafficking of GLUT4-containing vesicles to the surface in insulin-responsive metabolic tissues, such as, skeletal muscle and adipose tissue (Boucher et al. 2014, Czech 2017, Haeusler et al. 2018). In contrast, immune cells predominately rely on GLUT1 for basal and activation-induced glucose uptake (Freemerman et al. 2014, Macintyre et al. 2014). Unlike GLUT4, GLUT1 regulation is largely regulated at the transcriptional level rather than through rapid insulin-dependent trafficking (Macintyre et al. 2014). Parallelly, insulin receptor activation stimulates the MAP kinase (MAPK) signaling pathway, which primarily mediates insulin mitogenic effects (Worster et al. 2012). In metabolic disorders, reduced insulin sensitivity disrupts the integrated regulation of metabolic and immune signaling. As tissue becomes less responsive to insulin, pancreatic β cells compensate by enhancing insulin secretion to maintain normal glucose levels, resulting in hyperinsulinemia. Under conditions of chronic hyperinsulinemia and insulin resistance, insulin receptor signaling through the PI3K-Akt pathway becomes selectively impaired, whereas, signaling through the MAPK-ERK pathway is relatively preserved (Jiang et al. 1999, Cusi et al. 2000). This preferential activation of MAPK signaling cooperate with and amplify NF-κB activity, promoting the transcription of proinflammatory genes (Kracht et al. 2020, Jin et al. 2023). Concurrently, nutrient excess promotes the expansion and dysfunction of adipose tissue leading to adipocyte hypertrophy and induces adipose tissue stress. The resulting stress leads to the release of danger associated molecular patterns, which activate NF-κB pathway and further perpetuate chronic inflammation. Consequently, these initial events induce the recruitment of circulatory monocytes into the adipose tissue, where they differentiate into classically activated macrophages. These macrophages secrete proinflammatory cytokines, such as TNFα, IL-1α, and monocyte chemoattractant protein-1 (MCP-1), which further amplify local and systemic inflammation (Weisberg et al. 2003, Hotamisligil 2006, Lumeng et al. 2007). Chronic hyperglycemia also suppresses key TCA cycle enzymes including, succinate dehydrogenase, stalling TCA cycle and accumulation of metabolites such as succinate (Stratmann et al. 2022, Fernández-Veledo et al. 2024). Elevated succinate together with free radicals, promotes the activation of the NLRP3 inflammasome, a multiprotein complex that facilitates the maturation and release of IL-1β, augmenting inflammatory responses (Tannahill et al. 2013, Dai et al. 2017, Wan et al. 2022).

Metabolic diseases are also associated with dysregulated amino acids metabolism, which is closely linked to chronic low-grade inflammation. Elevated branched chain amino acids (BCAAs), including leucine, isoleucine, and valine, contribute to inflammation in metabolic diseases because of impaired catabolism and their subsequent accumulation. Metabolic studies have shown that increased levels of BCAA and their metabolic byproducts, such as medium- and long-chain acylcarnitine, distinguish obese individuals with insulin resistance from those who remain insulin sensitive (Newgard et al. 2009). Notably, increased circulating BCAA levels are observed not only in patients with type 2 diabetes (T2D) but also prior to disease onset, indicating a potential role in disease onset (Wang et al. 2011, McCormack et al. 2013, Wang et al. 2017). Consistent with these observations, several studies demonstrate that dietary reduction of BCAA intake or pharmacological enhancement of catabolism improves insulin sensitivity, enhances energy expenditure, and ameliorates metabolic dysfunction (Xiao et al. 2014, Cummings et al. 2018, Yadao et al. 2018, Karusheva et al. 2019). Importantly, the accumulation of BCAAs activates mTOR signaling, which impairs the insulin signaling pathway and contributes to insulin resistance (Lynch and Adams 2014). mTOR activation also enhances NF-κB activity, promoting proinflammatory cytokine release and chronic low-grade inflammation (Dai et al. 2019, Xu et al. 2021). In addition, elevated BCAA levels act as a metabolic signal that promotes inflammasome assembly, leading to IL-1β maturation and release, thereby sustaining the chronic inflammation (Haggadone et al. 2025). Beyond BCAAs, dysregulated tryptophan metabolism has also emerged as a key contributor to inflammation and immune dysfunction in metabolic diseases. In obese individuals, circulating tryptophan levels are reduced due to increased activation of IDO1 (Wolowczuk et al. 2012, Mallmann et al. 2018, Cussotto et al. 2020). The increase enzymatic activity of IDO in diabetes depletes tryptophan and enhances the production of kynurenine pathway metabolites, including 3-hydroxykynurenine (Favennec et al. 2015, Kozieł and Urbanska 2023). The increase in these metabolites corelate with macrophage M1 polarization, which sustain proinflammatory function (Teunis et al. 2025). High fat diet supplemented with tryptophan or tryptophan-derived metabolite improves insulin sensitivity, inhibit proinflammatory macrophages, and promote antiinflammatory polarization of macrophages (Campos Zani de et al. 2022, Liu et al. 2025). Thus metabolic-immune cross-talk fuels chronic low-grade inflammation characteristics of metabolic disorders.

### Immune dysfunction and bacterial persistence in metabolic disorders

Metabolic disorders significantly impact immunometabolism and reprogram immune cells into a dysfunctional state that markedly heighten infection susceptibility. Multiple large cohort studies have consistently shown that metabolic diseases are linked with significantly higher risks of infection-related hospitalizations and mortality. In a large community-based cohort from Taiwan, individuals diagnosed with diabetes had an ~1.59-fold higher risk of hospitalization and a 1.71-fold increased risk of infection-related death when compared with people without diabetes (Chang et al. 2019). Moreover, the risk of both morbidity and mortality was notably elevated at fasting plasma glucose levels exceeding 200 mg/dl (Chang et al. 2019). Similarly, a UK primary care-based cohort study reported markedly increased incidence rate ratio for infection-related hospitalization of 3.71 for type 1 diabetes and 1.88 for T2D compared with matched controls. In this analysis, diabetes was estimated to account for 6% of infection-based hospitalization and 12% of infection-related deaths (Carey et al. 2018). Observational data from large cohorts of people with obesity and dyslipidemia further highlighted that metabolic syndrome is linked to higher rates of infections and increased infection-related mortality (Holt et al. 2024, Carey et al. 2025b, Rao et al. 2025). These associations appear consistently across multiple population-based studies and are attributed to combined effects of insulin resistance and immune metabolic dysfunction.

In metabolic diseases, chronic nutrient excess overwhelms cellular metabolic capacity and drives insulin resistance. Elevated circulating glucose and lipids promote intracellular accumulation of lipid intermediates such as diacylglycerol (DAG) and ceramide. These intermediates activate stress- and lipid-sensitive kinases including JNK, IKKβ, and PKC, which inhibit IRS proteins and block the canonical PI3K-Akt signaling. Defective PI3K-Akt signaling disrupts cytoskeleton rearrangement and pseudopod formation, directly impairing phagocytosis in macrophages and neutrophils (Lv et al. 2019, Pan et al. 2022). *In vitro* experiments confirm that high glucose concentrations of 200-800 mg/dl significantly reduce phagocytosis of bacteria such as *S. aureus, Staphylococcus epidermidis*, and *Escherichia coli* (Oss Van 1971). Consistent with these findings, macrophages and neutrophils isolated from people with diabetes show similar defects in phagocytosis against multiple bacteria (Chanchamroen et al. 2009, Lecube et al. 2011, Restrepo et al. 2014). Hyperglycemia further worsen immune function by accelerating the formation of advanced glycation end products (AGEs). AGEs modify cell surface receptors (Zhang and Swaan 1999), opsonins (Singh et al. 2014), and cytoskeletal proteins (Lan et al. 2015, Sonawane et al. 2021), thereby blocking pathogen recognition, receptor signaling, and phagosome formation. In addition, binding and uptake of AGE induces apoptosis in macrophages, further compromising their phagocytic function (Gao et al. 2017). Beyond their direct effects on immune cells, AGEs accumulate in extracellular matrix where they crosslink structural proteins such as collagen and elastin, causing tissue stiffness. This stiff matrix hinders immune cell trafficking to the site of infection (Haucke et al. 2014).

Dysregulated amino acid metabolism in metabolic disorders impairs immune defense mechanisms, promoting bacterial persistence and immune evasion. Elevated levels of BCAA, negatively regulates autophagy by inhibiting autophagosome formation, disrupting a key cellular mechanism for degrading intracellular pathogens (Zhang et al. 2016, Perveen et al. 2025). Autophagy plays a crucial role in innate immune defense mechanism by targeting invading bacteria. While suppression of autophagy allows pathogens such as *M. tuberculosis* and *Salmonella* to evade clearance, the direct impact of BCAA-mediated autophagy inhibition on their intracellular survival remains to be determined. Notably, certain bacteria such as *L. monocytogenes* and *S. aureus* uptake excess BCAA and converts them into branched-chain fatty acids (BCFAs) (Sun and O’Riordan 2010, Kaiser et al. 2016, Kaiser and Heinrichs 2018). These BCFAs are subsequently embedded into the bacterial cell membrane, altering its fluidity and permeability, which limits the ability of antimicrobial peptides to disrupt bacterial membranes (Sun et al. 2012, Kaiser et al. 2016, Chowdhury et al. 2025a). Metabolic diseases also skew arginine metabolism by increasing arginase activity, which limits substrate availability for NOS (Kashyap et al. 2007). This reduces NO production, impairing the ability of immune cells to eliminate the bacterial pathogens. At the same time, arginine switch increases polyamine synthesis, promoting bacterial growth while further subverting the immune response. For example, *S. typhimurium* uses polyamines to assemble T3SS-1, which enables the bacteria to invade the host cells (Miki et al. 2024). Similarly, *H. pylori* exploit polyamines to facilitate gastric colonization and propel the oxidative-stress mediated progression of gastric cancer (Gobert and Wilson 2016, McNamara et al. 2024). Finally, dysregulated tryptophan metabolism can promote immunosuppression, creating conditions that promote the overgrowth and persistence of infections, including those caused by *M. tuberculosis* (Gautam et al. 2018).

Lipid abnormalities in metabolic diseases further impair immune function, allowing bacteria to persist and evade clearance. For example, elevated levels of circulating cholesterol can accumulate in plasma membranes, particularly within lipid rafts. The buildup alters membrane fluidity, making the membranes less fluid and more rigidly packed. These changes interfere with the normal clustering and signaling of membrane proteins that are essential for chemotaxis and phagocytosis. Therefore, immune cells show reduced ability to migrate toward the site of infection and become less effective in engulfing bacteria, which compromises the overall process of bacterial clearance (Qin et al. 2006). Hyperlipidemia further promotes excessive lipid uptake by macrophages, leading to lipid droplet accumulation and foam cell formation. These foam cells exhibit reduced phagocytic activity and impaired phagolysosomal maturation. Specifically, cholesterol overload prevents lysosome acidification, while cholesterol crystals in macrophage-derived foam cells induce lysosomal damage releasing their contents into the cytosol (Cox et al. 2007, Rajam̈aki et al. 2010, Lieberman and Swanson 2018). Together, these defects impede the effective clearance of engulfed bacteria. Notably, bacteria like *Chlamydia pneumoniae* and *Porphyromonas gingivalis* can actively promote foam cell formation, creating a lipid-rich intracellular environment that favors bacterial persistence (Giacona et al. 2004, Cao et al. 2007, Chen et al. 2008a, Yang et al. 2021). Additionally, excess FFAs and cholesterol activate mTOR, which suppresses autophagy and reduces autophagosome formation further limiting bacterial clearance (Jarocki et al. 2024). Collectively, nutrient excess compromise’s immune function, heighten susceptibility to infection and promotes pathogen persistence.

### ROS generated during metabolic disorders promotes bacterial persistence and biofilm formation

Molecular oxygen is fundamental to most life on Earth as it facilitates aerobic respiration to efficiently generate ATP. Its high redox potential makes oxygen an excellent electron acceptor, allowing cells to generate substantial amounts of energy (Rolfe and Brown 1997, Babcock 1999). In addition to its role in immunometabolism, oxygen also functions as a signaling molecule, mainly by promoting the production of ROS (Bartz and Piantadosi 2010). At low levels, ROS regulate cell growth and differentiation and play a crucial role in host defense against microbes (Thannickal and Fanburg 2000, Sies and Jones 2020). To maintain these beneficial effects, their physiological levels are tightly controlled through localized and spatially compartmentalized production, as well as the engagement of various ROS-neutralizing systems. However, when ROS production exceeds its neutralization, oxidative stress arises, leading to significant molecular damage (Schieber and Chandel 2014, Raghunath et al. 2018). In mammals, the seven isoforms of NADPH oxidases (NOX) are the primary enzymatic sources of ROS. NOX enzymes catalyse the one-electron reduction of molecular oxygen using NADPH as an electron donor to produce superoxide anion (O_2_^−^), which is subsequently converted into hydrogen peroxide (H_2_O_2_) (Bedard et al. 2007). In metabolic diseases, the mechanisms that regulate NOX expression and function are often perturbed, leading to excessive production of ROS. For example, both people with diabetes and animal models of diabetes show increased expression of NOX subunits (Nakayama et al. 2005, Huang et al. 2011). In addition, elevated glucose levels enhance NOX activity via DAG-mediated activation of protein kinase C (PKC) (Volpe et al. 2018). High glucose also accelerates the formation of AGEs, that bind receptor for advanced glycation end products receptor and further potentiate NOX activity (Tan et al. 2007). Similarly, in individuals with dyslipidemia elevated FFAs, such as palmitic acid, directly promote ROS production by activating NOX enzymes (Syed et al. 2010). Together, these alteration in NOX expression and activity leads to excessive ROS production, which disrupts the overall redox balance.

ROS play dual role in bacterial persistence and biofilm formation. While ROS can eliminate bacteria, they can also contribute to bacterial persistence by triggering stress responses, activating protective efflux pumps that increases antibiotics tolerance, and driving a subpopulation of bacterial cells into a slow-growing, low-metabolic state. To survive ROS-induced stress, bacteria have evolved specialized transcriptional regulators that sense oxidative stress and trigger the expression of genes responsible for antioxidant defenses and ROS-neutralizing mechanisms. For example, in *Pseudomonas aeruginosa*, OxyR primarily senses and responds to H_2_O_2_ (Heo et al. 2010). Once activated, these regulators induce expression of antioxidant genes encoding catalases, peroxidases, and other oxidative stress-related proteins that mitigate oxidative damage (Heo et al. 2010, Wei et al. 2012). Oxidative stress also actives multidrug efflux systems that contribute to antibiotic tolerance. For example, oxidative stress induces the AcrAB-TolC efflux pump through the redox sensing SoxRS regulon in *Klebsiella pneumoniae* (Anes et al. 2021). The upregulation of AcrAB-TolC enhances the efflux of antibiotics such as fluoroquinolones, directly linking ROS exposure to increased antibiotic tolerance in Gram-negative pathogens (Baucheron et al. 2004). In addition, the SoxRS regulon controls expression of small regulatory RNA *micF*, which represses translation of the outer membrane protein OmpF (Chou et al. 1993). Reduced OmpF expression limits antibiotic entry, further enhancing antimicrobial tolerance (Chou et al. 1993, Rosas and Lithgow 2022). Similarly, oxidative stress in *S. aureus* is sensed by the transcriptional regulator MgrA and its functional homologue SarZ, which function as thiol-based regulatory systems controlling genes involved in virulence, the oxidative stress response, and antibiotic resistance(Chen et al. 2006, 2008b). MgrA regulates the multidrug efflux pumps including *norA, norB*, and *tet38*, which modulates the resistance to fluoroquinolones, tetracycline, and other biocidal compounds (Truong-Bolduc et al. 2005, Chen et al. 2006). Beyond these regulatory mechanisms, oxidative stress overlap with dormancy or senescence-like state. For example, ROS-induced carbonylation of amino acid residues misfolds proteins that inactivate metabolic and replication enzymes, slowing bacterial growth (Maisonneuve et al. 2008). ROS can reduce the proton motive force, which suppresses the metabolic activity and favors the formation of persister populations (Conlon et al. 2016). In addition, H_2_O_2_ and O_2_^−^ oxidize DNA bases causing single-strand breaks that trigger bacterial DNA damage response including the SOS response, which is associated with growth arrest and antibiotic tolerance (Podlesek and Žgur Bertok 2020). Collectively, these adaptive and damage-induced responses enable bacteria to survive and persist during infection, particularly in hyperglycemic wounds where oxidative stress is chronically elevated.

Many ROS defense mechanisms are also closely linked to biofilm formation and motility, further enhancing bacterial persistence in metabolic disorders, as summarized in Table 1. In many bacteria, the second messenger cyclic di-GMP (c-di-GMP) plays a central role in directing the transition from a planktonic to biofilm lifestyle. Chronic exposure to ROS, such as H_2_O_2_ can select for bacterial variants that possess an enhanced capacity to form biofilms. For example, in *P. aeruginosa*, long-term exposure to H_2_O_2_ selects for small-colony variants carrying mutations in *wspF*, leading to elevated intracellular c-di-GMP levels and increased biofilm formation (Chua et al. 2016). Oxidative stress also regulates bacterial motility and attachment, key steps in biofilm initiation (Strempel et al. 2017). In *Salmonella*, the SoxS regulon inhibits bacterial motility by suppressing genes involved in flagellar assembly (Thota et al. 2020). By inhibiting flagellar motility, bacteria shift toward a surface-associated lifestyle. In *Acidovorax citrulli*, this transition is aided by OxyR, which promotes surface attachment by inducing the expression of genes involved in adhesion (Wang et al. 2022). ROS-driven signaling also plays a critical role in regulating extracellular polymeric substances (EPS) production, that is important for the formation of mature biofilm (Villa et al. 2012). EPS form the structural scaffold of biofilms which in turn provide protection from oxidative stress. In *P. aeruginosa*, ROS mediated regulation of EPS is tightly linked to the emergence of the mucoid phenotype during chronic infection. Specifically, H_2_O_2_ stimulates alginate biosynthesis, leading to increased EPS production and conversion from a nonmucoid to the mucoid state (Mathee et al. 1999, Ramsey and Wozniak 2005, Limoli et al. 2014, Tan et al. 2018). This alginate-rich matrix not only stabilizes mature biofilms but also enhances resistance to ROS, antibiotics, and host immune response (Simpson et al. 1989, Walters et al. 2003, Malhotra et al. 2018). Thus ROS-induced alginate production in *P. aeruginosa* represents an adaptive response that promotes long-term persistence, particularly in metabolically compromised tissues characterized by sustained oxidative stress.

### Hypoxia-driven bacterial persistence and biofilm formation in metabolic disorders

Hypoxia, defined as low oxygen levels, is a common feature of many metabolic diseases (Lefere et al. 2016, Gonzalez et al. 2018, Luo et al. 2022). It arises from multiple interconnected factors, including vascular damage, reduced oxygen-carrying capacity of hemoglobin due to glycation, structural defects in red blood cells, and damage to lung membranes that impair oxygen diffusion (Bunn et al. 1978, Babu and Singh 2004, Rask-Madsen and King 2013, Rajasurya et al. 2020). In addition, increased oxygen demand resulting from elevated metabolic activity further contributes to low-oxygen conditions (Wang et al. 2005). In response to hypoxia, the body has evolved multiple hypoxia-sensing mechanisms to restore oxygen homeostasis. Central to this response is hypoxia-inducible factor (HIF), a transcription factor that regulates cellular adaptation to low oxygen conditions. HIF functions as a heterodimer consisting of an oxygen sensitive α-subunit and a constitutively expressed β-subunit. In vertebrates, three isoforms of the HIFα subunit have been identified—HIF1α, HIF2α, and HIF3α. Among these, HIF1α and HIF2α are the most extensively studied and serve as key regulators of hypoxia-responsive gene expression. In contrast, HIF3α exists in several alternatively spliced variants, some of which act as negative regulators of HIF1α and HIF2α (Ziello et al. 2007, Semenza 2012, Yuan et al. 2023). Moreover, HIFα stability and activity are mainly controlled by oxygen-sensing enzymes, called prolyl hydroxylase domain (PHDs) proteins (Epstein et al. 2001, Ivan et al. 2001). Three PHD isoforms, PHD1, PHD2, and PHD3 have been identified, each with distinct tissue distribution and regulatory functions (Bruick and McKnight 2001, Fong and Takeda 2008). Under normal oxygen conditions, PHD enzymes use iron and α-ketoglutarate to hydroxylate HIFα at two specific proline residues (Jaakkola et al. 2001, Hausinger 2004). This modification marks HIFα for recognition by the E3 ubiquitin ligase von Hippel–Lindau (VHL) protein, leading to its ubiquitination and proteolysis (Ivan et al. 2001, Jaakkola et al. 2001). However, under hypoxia, low oxygen inhibits PHD activity and prevents HIFα hydroxylation. Under these conditions, HIFα stabilizes, accumulates in the cell, and translocate to the nucleus, where it regulates the transcription of hypoxia-responsive genes (Semenza et al. 1996, Ebert and Bunn 1999). Additionally, factor inhibiting HIF (FIH), another oxygen-dependent enzyme provides additional layer of regulation for HIF. FIH hydroxylates an asparagine residue within the C-terminal transactivation domain of HIFα. This modification prevents the recruitment of transcriptional coactivators p300/CBP, suppressing HIF transcriptional activity (Lando et al. 2002). Together, PHDs and FIH tightly regulate HIF signaling and enable cells to adapt to hypoxia.

In metabolic diseases, such as diabetes, tissues are often low in oxygen, yet high glucose impairs the stabilization and activity of HIF1α, preventing an appropriate hypoxic response (Catrina et al. 2004, Ramalho et al. 2017). Hyperglycemia promotes proteasomal degradation of HIF1α through the canonical PHD–VHL pathway (Zheng et al. 2022), while additional noncanonical mechanism that involves HIF1α interaction with Hsp90, may also contribute (Liu et al. 2007, García-Pastor et al. 2019). In addition to hyperglycemia, dyslipidemia further undermines hypoxia signaling. Elevated levels of low-density lipoprotein reduce HIF1α and HIF2α protein levels, limiting the ability of tissues to adapt to hypoxia in individuals with hyperlipidemia (Shao et al. 2021). Moreover, build-up of fatty acids alters cellular metabolism and reduces the accumulation of TCA cycle intermediates such as succinate and fumarate. Because these metabolites normally inhibit PHD activity under hypoxia conditions, their depletion sustains PHD function even during hypoxia, further enhancing HIF1α degradation (Hewitson et al. 2007). Beyond PHD-dependent regulation, high glucose metabolism leads to the accumulation of methylglyoxal (MGO), a reactive dicarbonyl by-product of glycolysis that disrupts HIF signaling. MGO modifies HIF1α through the glycation of lysine and arginine residues, compromising its stability and transcriptional activity during hypoxia. This modification increases HIF1α association with molecular chaperones Hsp40 and Hsp70, which in turn promotes recruitment of the chaperone-dependent E3 ubiquitin ligase C-terminus of Hsp70-interacting protein. This process drives ubiquitination and proteasomal degradation of HIF1α through a PDH–VHL independent pathway (Bento et al. 2010). In addition to altering HIF1α stability, MGO accumulation also directly inhibits the transcriptional activity of HIF1. MGO modifies HIF1α and interferes with its heterodimerization with HIF1β, thereby reducing its binding to hypoxia-responsive elements in HIF target genes (Ceradini et al. 2008). Furthermore, MGO modifies the transcriptional coactivator p300, hindering its interaction with HIF1α and further diminishing HIF-dependent gene expression (Thangarajah et al. 2009). Collectively, these mechanisms converge to suppress HIF signaling in hyperglycemic and dyslipidemia conditions, even when the underlying tissues are hypoxic.

Hypoxia induces an adaptive response against infection by turning on the expression of genes, such as NOS2, antimicrobial peptides, and proinflammatory cytokines to boost antibacterial defense (McGettrick and O’Neill 2020). Yet, long-term hypoxia in metabolic diseases impairs immune cell function and compromises antimicrobial defense, promotes persistent inflammation, and delays tissue repair. For example, low oxygen levels blunt neutrophil migration, an effect that is further aggravated by hyperglycemia in diabetes (Rotstein et al. 1988, Roy et al. 2022). Additionally in neutrophils, hypoxia stabilizes HIF1α, which activates NF-κB signaling to upregulate the expression of antiapoptotic genes, while suppressing pro-apoptotic pathways and inhibiting caspase activation, promoting neutrophil survival (Walmsley et al. 2005). Although increased survival may initially support host defense, hypoxia restricts NOX-dependent respiratory burst activity, reducing effective bacterial killing despite prolonged neutrophil presence (McGovern et al. 2010). Moreover, both hypoxia and hyperglycemia independently promote the release of neutrophil extracellular traps (NET) via the process of NETosis (Wong et al. 2015, Ye et al. 2025). NETs are composed of decondensed chromatin decorated with histones and antimicrobial proteases that trap and kill extracellular bacteria (Stapels et al. 2014, Papayannopoulos 2018, Doolin et al. 2020). While acute NETosis is a short-term innate response against pathogens, chronic NETosis induced by hypoxic–hyperglycemic conditions leads to sustained inflammation, cytotoxicity, and tissue damage. For example, NETs and extracellular histones can exert cytotoxic effects on the epithelial and endothelial cells (Saffarzadeh et al. 2012). In diabetic wounds, excessive NET formation releases neutrophil elastase and other proteases that degrade the extracellular matrix and impair wound healing (Wong et al. 2015). These dysregulated NETs also activate platelets and the coagulation pathway, causing significant vessel blockage and reduced tissue perfusion (Shafqat et al. 2022). However, NET-mediated antimicrobial defense is often compromised under these conditions, as *S. aureus*, which is abundant in diabetic wounds, produces DNases that degrade NETs to escape capture (Berends et al. 2010). Like neutrophils, macrophage function is also profoundly impaired under hypoxic and hyperglycemic conditions. Low oxygen tension and metabolic stress limits macrophage plasticity and locks them into a persistent proinflammatory state (Morey et al. 2019). Their failed transition to antiinflammatory state limits proresolving capacity, which is essential for tissue repair and regeneration (Mills et al. 2000, Wynn and Vannella 2016). In addition, hypoxia and hyperglycemia diminishes macrophage phagocytosis capacity by lowering the expression of CD36 and SR expression, further leading to the persistence of bacteria in metabolic diseases (Morey et al. 2019).

Beyond causing immune dysfunction, the chronic hypoxia in metabolic diseases also directly favors pathogen survival. Many facultative anaerobic bacteria adapt to hypoxic environment by switching from aerobic to anerobic metabolism. This switch is largely controlled by the fumarate and nitrate reduction regulator (FNR), a global transcriptional regulator that senses oxygen availability (Spiro 1994, Crack et al. 2004, Green and Paget 2004, André et al. 2021). In *Shigella flexneri*, FNR allows adaptation to anaerobic conditions by regulating both metabolic and virulence factors, thereby promoting survival in the intestinal environment. FNR represses expression of *spa32* and *spa33*, which are key regulators of T3SS. This repression yields a partially functional T3SS with longer needles and reduced substrate secretion, that attenuates virulence and favors colonization over invasion (Marteyn et al. 2010). Loss of *fnr* significantly impairs the ability of *S. flexneri* to colonize the host in an *in vivo* infection model (Marteyn et al. 2010). FNR also plays an important role in the intestinal colonization of several other enteric pathogens, such as *E. coli* and *S. enterica*. In these bacteria, FNR functions as global regulator that facilitates adaption to the anaerobic environment in the host intestine by controlling the expression of genes involved in metabolism, virulence, and colonization (Fink et al. 2007, Crofts et al. 2018). Notably, metabolic diseases significantly increase the risk and severity of infections caused by enteric pathogens (Thaiss et al. 2018, Yao et al. 2018). Similarly, in *P. aeruginosa*, the functional homolog of FNR is the anaerobic regulator Anr, which enables the bacteria to sense oxygen and regulate gene expression in response to oxygen availability (Zimmermann et al. 1991). Anr coordinates the bacterial adaptation to anaerobic conditions by regulating pathways involved in denitrification, arginine fermentation, and oxidative stress responses (Hammond et al. 2015). This function is especially important in environments such as cystic fibrosis lung, where thick mucus creates hypoxic niches and in diabetic wounds, where poor vascularization limits oxygen diffusion. While hyperglycemia broadly enhances *P. aeruginosa* virulence, it remains unclear, whether Anr contributes to this enhanced virulence under hypoxic diabetic conditions. Overall, these hypoxia-sensing systems enable bacteria to adjust their metabolism and successfully colonize oxygen-limited tissues.

Hypoxia both initiates and amplifies bacterial biofilm formation by creating a persistently low-oxygen niche (Table 1). Chronic hypoxia in metabolic diseases creates an environment that favors biofilm-associated infections, making it difficult to eradicate. Nearly one in five patients with diabetic foot infections develop underlying osteomyelitis, which significantly raises the risk of lower limb amputation (Eneroth et al. 1999). In skeletal tissue, which is inherently hypoxic, *S. aureus* osteomyelitis further depletes local oxygen levels, amplifying hypoxia and promoting biofilm formation (Urish and Cassat 2020). Similarly, after craniotomy, *S. aureus* can form biofilms on the bone flap within a hypoxic microenvironment (Roy et al. 2024). Consistent with these observations, *in vitro* studies show that low oxygen conditions enhance *S. aureus* biofilm formation by upregulating biofilm-associated genes (Cramton et al. 2001). Reduced oxygen bioavailability also limits bacterial respiration, which can trigger cell lysis and the release of extracellular DNA, further strengthening the biofilm scaffold (Mashruwala et al. 2017). Similar hypoxia-driven biofilm responses have been observed in cystic fibrosis lungs, where thick and sticky mucus restricts oxygen flow and *P. aeruginosa* predominately exists in the biofilm state (Singh et al. 2000, Worlitzsch et al. 2002). *Pseudomonas aeruginosa* is also frequently isolated from diabetic foot ulcers, where hypoxic wound environment is strongly correlated with the formation of biofilms (Garousi et al. 2023). In parallel, hypoxia-induced metabolic reprogramming of immune cells in infected tissues leads to elevated levels of the immunometabolite itaconate, driven largely by HIF1α-dependent *Irg1* induction (Li et al. 2022c). Itaconate is abundant in infected lungs, where it can induce membrane stress in *P. aeruginosa* and stimulates EPS production. The newly synthesized EPS can further prompt itaconate production by immune cells, creating a self-sustaining cycle that promotes persistent biofilm formation (Riquelme et al. 2020). Host-derived itaconate alters *S. aureus* metabolism, shifting bacterial resources toward EPS production and promoting biofilm formation. Specifically, during *S. aureus* infection, staphylococcal glycolytic activity induces mitochondrial stress in host immune cells, which upregulates IRG1 and increases itaconate production. Itaconate in turn inhibits *S. aureus* glycolysis, creating selective pressure for metabolic adaptation. This adaptation redirects carbon flux to derive EPS synthesis and biofilm formation. (Tomlinson et al. 2021). The itaconate pathway is also relevant in diabetes, although its precise contribution to biofilm formation in diabetic tissue remains unclear. Overall, these observations support a positive association between chronic hypoxia in metabolic diseases and enhanced bacterial biofilm formation.

### Pathogen exploitation of host nutrient excess in metabolic diseases

**Figure 2:**
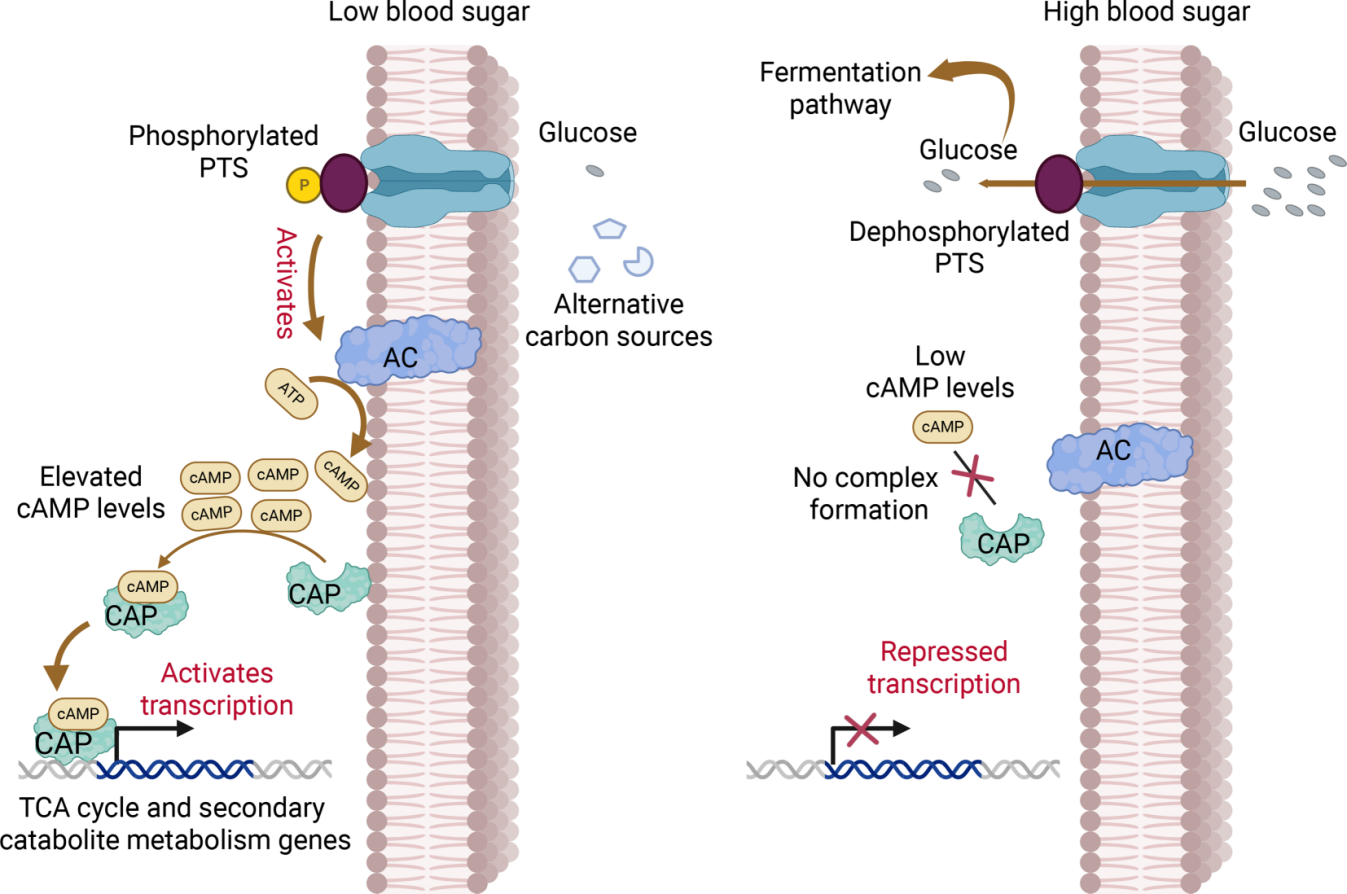
Glucose-dependent carbon catabolite regulation in Gram-negative bacteria. In Gram-negative bacteria, when glucose levels are low, bacteria switch from using glucose to using alternative carbon sources. Under these conditions, the phosphotransferase system (PTS) remains phosphorylated and activates adenylate cyclase (AC), which converts ATP into cyclic AMP (cAMP), leading to increased intracellular cAMP levels. cAMP then binds to CAP, forming cAMP–CAP complex. This complex activates the transcription of genes involved in the TCA cycle and the metabolism of alternative carbon sources (left). In contrast, under high glucose conditions, the PTS is predominately dephosphorylated and actively transports glucose into the cell. In this state, it does not activate AC, leading to low intracellular cAMP levels. Consequently, the cAMP–CAP complex does not form, and the expression of genes involved in the TCA cycle and alternative carbon source utilization remains repressed. Instead, glucose metabolism is directed toward fermentative pathways, enabling ATP production without complete oxidation of glucose (right). Created in https://BioRender.com.

Bacteria exploit insulin resistance in infected tissue by taking advantage of the resulting nutrient-rich microenvironment that favors pathogen survival and replication. For example, *E. coli, B. subtilis, S. aureus*, and *Streptococcus mutans* frequently engage in overflow metabolism, rapidly consuming excess glucose and producing fermentative by-products like lactate, acetate, ethanol, and acetoin (Sonenshein 2007, Zhuang et al. 2011, Chen et al. 2020, Chowdhury et al. 2025a, Shahreen et al. 2025). This metabolic shift occurs in part because glucose oxidation generates acetyl-CoA at a rate that exceeds the processing capacity of the TCA cycle. Consequently, carbon flux is diverted away from complete oxidation and towards fermentation pathways (Fig. 2). This metabolic strategy resembles the Warburg effect observed in host cells, in which glucose is not completely oxidized through the TCA cycle despite the presence of oxygen favoring rapid energy generation over optimal energetic efficiency (Passalacqua et al. 2016, Chowdhury et al. 2025b). Beyond intrinsic metabolic constraints, TCA cycle activity is further suppressed under hyperglycemia by carbon catabolite repression (CCR), a regulatory mechanism that prioritizes glucose utilization over oxidative metabolism and alternative carbon source usage (Tobisch et al. 1999, Görke and Stülke 2008, Fujita 2009). CCR operates through distinct molecular pathways in Gram-negative and Gram-positive bacteria (Görke and Stülke 2008). In Gram-negative bacteria, high glucose availability results in decreased intracellular levels of cyclic-AMP (cAMP) and prevents the formation of cAMP–catabolite activator protein (CAP) complex. This complex is required to activate transcription of TCA cycle genes and secondary carbon source metabolism (Feucht and Saier 1980, Notley-McRobb et al. 1997, Kremling et al. 2015) (Fig. 2). In Gram-positive bacteria, CCR is mediated by the phosphocarrier protein, HPr, which upon phosphorylation interacts with catabolite control protein A (CcpA). The HPr–CcpA complex binds to catabolite-responsive elements, directly repressing transcription of genes encoding TCA cycle enzymes and operons involved in alternative carbon metabolism (Deutscher et al. 1995, Jones et al. 1997, Tobisch et al. 1999). Under hyperglycemic conditions, elevated levels of glycolytic intermediates lead to the enhanced Hpr phosphorylation, which in turn results in an increased CcpA-mediated CCR (Deutscher et al. 1995). Additionally, the accumulation of overflow metabolites promotes metabolic cooperation among pathogens within infected tissues. For example, in diabetic wounds, *S. aureus* frequently coexists with *P. aeruginosa* (DeLeon et al. 2014, Macdonald et al. 2021, Dörr et al. 2023). The acetoin produced by *S. aureus* through overflow metabolism is scavenged by *P. aeruginosa* as a carbon source. This metabolic cross-feeding supports the survival and persistence of both pathogens in the infected niche (Camus et al. 2020). Moreover, overflow metabolism allows bacteria to reabsorb the fermentative by-products at later stages, providing metabolic flexibility as nutrient availability fluctuates during infection (Wolfe 2005).

Hyperglycemia also directly increases the virulence and pathogenic potential of many bacteria, increasing their ability to colonize host tissues and cause more severe infections. By utilizing carbon-sensitive regulatory systems, bacteria detect elevated glucose levels and turn on the expression of their virulence genes. For example, in *S. aureus*, increased glucose flux elevates intracellular ATP levels, which then activates the accessory gene regulator (*agr*) locus (Thurlow et al. 2020). The Agr system drives the expression of RNAIII and activates the response regulator AgrA, which together induce the expression of various virulence factors (Jenul and Horswill 2019, Cheung et al. 2021). Specifically, AgrA directly controls the transcription of phenol-soluble modulins α and β, while RNAIII releases the translational block and prompt the expression of α hemolysin and leukocidins (Novick et al. 1993, Morfeldt et al. 1995, Queck et al. 2008). The Agr system also promotes the expression of extracellular proteases such as the metalloproteinase aureolysin (Aur), the serine protease SspA, and the cysteine protease SspB (Oscarsson et al. 2006, Cheung et al. 2011, Jenul and Horswill 2019). These bacterial proteases break down host extracellular matrix proteins and facilitate tissue invasion and bacterial dissemination. Similarly, in *Streptococcus pneumoniae* hyperglycemia enhances capsule biosynthesis, introducing a protective outer polysaccharide layer (Yang et al. 2025). The capsule is a key virulence factor that protects the bacterium from opsonization and phagocytosis by host immune cells (Hyams et al. 2010). Additionally, host-derived fatty acids which are elevated in people with hyperlipidemia triggers *S. aureus* to activate its type VII secretion system (T7SS) (Lopez et al. 2017, Li et al. 2022c, Jiang et al. 2025). Specifically, bacterial fatty acid kinase complex phosphorylates these fatty acids and incorporates them into the membrane, reducing its fluidity. This signals *S. aureus* to elevate T7SS, helping the bacteria to survive in the bloodstream and abscesses (Lopez et al. 2017, Bowman and Palmer 2021). Overall, excess nutrients activate various regulatory pathways that boosts virulence factors, leading to worse infection outcomes.

Chronic hyperglycemic conditions further impair infection clearance by promoting bacterial biofilms formation by pathogens such as *S. aureus, S. epidermidis*, and *P. aeruginosa*. These bacteria utilize excess glucose to enhance extracellular matrix production (Seidl et al. 2008, She et al. 2019), ensuring their longer persistence in diabetic infections (Table 1). For example, in *S. aureus* and *S. epidermidis* hyperglycemic conditions suppress the TCA cycle. This suppression increases the expression of the *icaADBC* operon, which is essential for the synthesis of polysaccharide intercellular adhesion (PIA), a critical component of *S. aureus* and *S. epidermidis* biofilm matrix. Consistent with this finding, genetic or pharmacological abrogation of TCA cycle increases the production of PIA (Vuong et al. 2005, Sadykov et al. 2008, Zhu et al. 2009). High glucose concentration also induces metabolic stress that leads to a significant drop in intracellular cyclic di-AMP (c-di-AMP) levels. The lower levels of c-di-AMP are associated with increased biofilm formation in *S. aureus* (Syed et al. 2024). Additionally, AGEs that accumulate under hyperglycemic conditions and are prevalent in diabetic wounds can enhance biofilm formation in *S. aureus*. AGEs upregulate alternative sigma factor B (*sigB*), which enhances extracellular DNA release and promotes biofilm formation (Table 1). Mutants lacking *sigB* do not respond to the presence of AGE confirming that *sigB* is essential for AGEs-dependent biofilm formation (Xie et al. 2020, Ni et al. 2024). A related mechanism is observed in *P. aeruginosa*, where exposure to very high levels of exogenous glucose raises intracellular glucose-6-phosphate levels. Glucose-6-phosphate activates NicD, a diguanylate cyclase that raises c-di-GMP levels (Park et al. 2021). Elevated c-di-GMP promotes biofilm formation by enhancing production of extracellular matrix components and reducing motility (Römling et al. 2005, Ha and O’Toole 2015, Hengge et al. 2016). In addition, high glucose also directly induces the expression of *pslA* (She et al. 2019), a key component of the *psl* operon, which is required for polysaccharide production in *P. aeruginosa* biofilms (Friedman and Kolter 2004, Jackson et al. 2004, Matsukawa and Greenberg 2004). Collectively, chronic hyperglycemia strengthens biofilms and bacterial survival in diabetic infections.

### Impact of metabolic disorders on antibiotic resistance

Metabolic diseases create a microenvironment that is marked by weakened immune responses and abundant nutrients for bacteria, which fosters more aggressive and invasive infections. As a result, long-term antibiotic treatment is often required to control these infections. Alarmingly, individuals diagnosed with diabetes have experienced a 60% rise in antibiotic prescription for lower respiratory infections (Venmans et al. 2009). In addition, epidemiological studies suggest that individuals with metabolic diseases experience higher rates of infection relapse. For example, the recurrence rate of urinary tract infection is higher in individuals with diabetes compared with nondiabetic individuals, reaching 24%–37% in diabetes in multiple cohort studies (Papp and Zimmern 2023). Therefore, the higher frequency of infections leads to more prevalent use of antibiotics and a greater likelihood of antibiotic treatment failure (Shallcross et al. 2017, Carrillo-Larco et al. 2022). Hyperglycemia in diabetic tissues may contribute to the expansion of antibiotic-resistant *S. aureus* by fostering a glucose-rich environment that supports rapid bacterial growth. Although hyperglycemia does not necessarily increase the baseline mutation rate, the resulting increase in bacterial population size raises the probability that resistance-conferring mutations will arise during replication. Once such mutations emerge, including those affecting *rpoB*, antibiotic pressure can allow them to dominate, particularly in diabetic tissues, where excess glucose provides a favorable growth environment for the expansion of resistant mutants. Furthermore, diabetic microenvironment may reduce the fitness cost associated with antibiotic resistance. Notably, clinical vancomycin-intermediate *S. aureus* strains, which are attenuated in nondiabetic mice, display enhanced growth and virulence during infection in diabetic mice (Shook et al. 2025). Concerningly, people with diabetes frequently depend on conventional antibiotics to treat infections, which can unintendedly increase the emergence of drug-resistant bacteria (Barwell et al. 2017, Mougakou et al. 2023).

**Figure 3:**
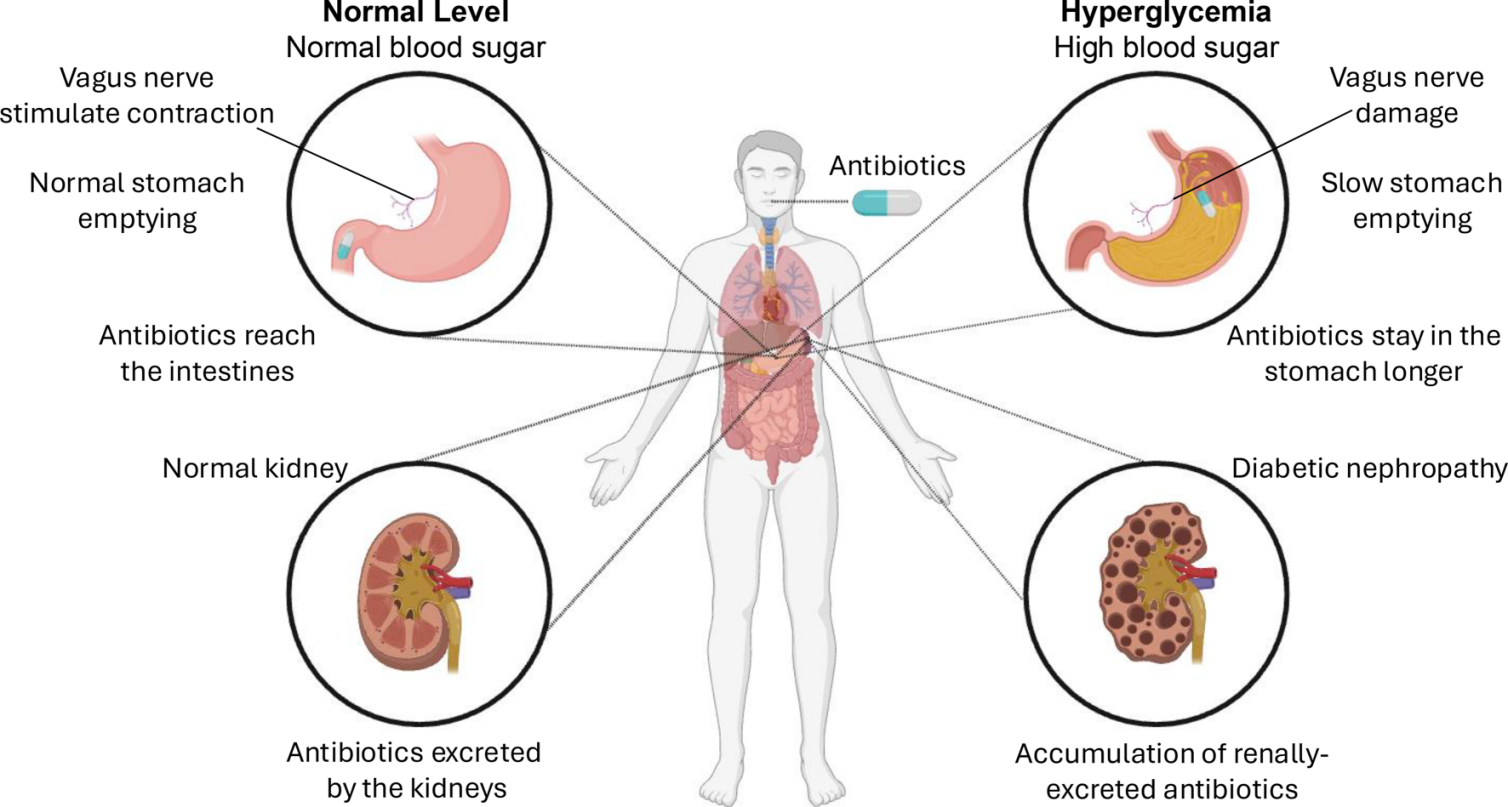
A link between metabolic dysfunction and antibiotic resistance. The vagus nerve stimulates stomach contractions that move food and orally consumed antibiotics from the stomach into the intestines (upper left). Diabetes damages the vagus nerve, which disrupts stomach contractions. This impairment causes delayed gastric emptying, resulting in the food and orally consumed antibiotics to stay in the stomach. The delay in absorption leads to a subtherapeutic antibiotics levels, which increase the risk of bacterial persistence and antibiotic resistance (upper right). The kidneys filter the blood and efficiently excrete waste along with many antibiotics in the urine (lower left). The chronic high blood sugar levels can cause diabetic nephropathy, damaging the kidney filtering units. The impaired kidney function reduces the ability to clear waste and drugs, including certain antibiotics, causing their accumulation and potential toxicity (lower right). Created in https://BioRender.com.

These therapeutic challenges are further complicated by the fact that metabolic syndromes alter the distribution of antibiotics in the body. Such conditions substantially modify antibiotic pharmacokinetics through multiple mechanisms, including delayed gastric emptying, impaired tissue perfusion, and altered hepatic and renal metabolism. Changes in gut motility, especially delayed gastric emptying, can slow the rate of absorption of orally administered drugs (Nimmo 1976, Shankar et al. 2024). Gastric emptying is a regulated process in which the stomach releases its content into the small intestine, which normally takes ~2–4 h for a meal (Banks et al. 2023). In metabolic diseases, such as diabetes this process is significantly delayed (Soykan et al. 1998, Aleppo et al. 2017, Camilleri et al. 2018). One major reason for this delay is that chronically high sugar over time damage the vagus nerve, which controls stomach muscle contractions and slow stomach emptying (Litwin 2024) (Fig. 3). Increased oxidative stress and chronic inflammation in diabetes further weakens the stomach muscle function (Kashyap and Farrugia 2011). In addition, hormonal imbalances also contribute to delayed gastric emptying in diabetes. Altered levels of gastrointestinal hormones such as glucagon-like peptide-1 (GLP-1) and ghrelin, disrupt the signaling pathways that regulate gastric motility and emptying (Nauck et al. 2021). This delay can significantly alter oral drug absorption, including antibiotics by keeping them longer in the stomach before they reach the intestines where most uptake occurs. Moreover, diabetes-related changes in gastric motility can alter the gut microbiota, promoting dysbiosis (Singh et al. 2021). The dysbiosis can further influence drug pharmacokinetics by affecting metabolism, reducing solubilization, and increasing binding to undigested fibers. Together, these factors can limit antibiotic bioavailability, leading to subtherapeutic plasma concentrations that may be insufficient to effectively eradicate the bacteria. Prolonged exposure to lower antibiotics levels further heightens the risk of selecting resistant bacterial strains.

**Figure 4:**
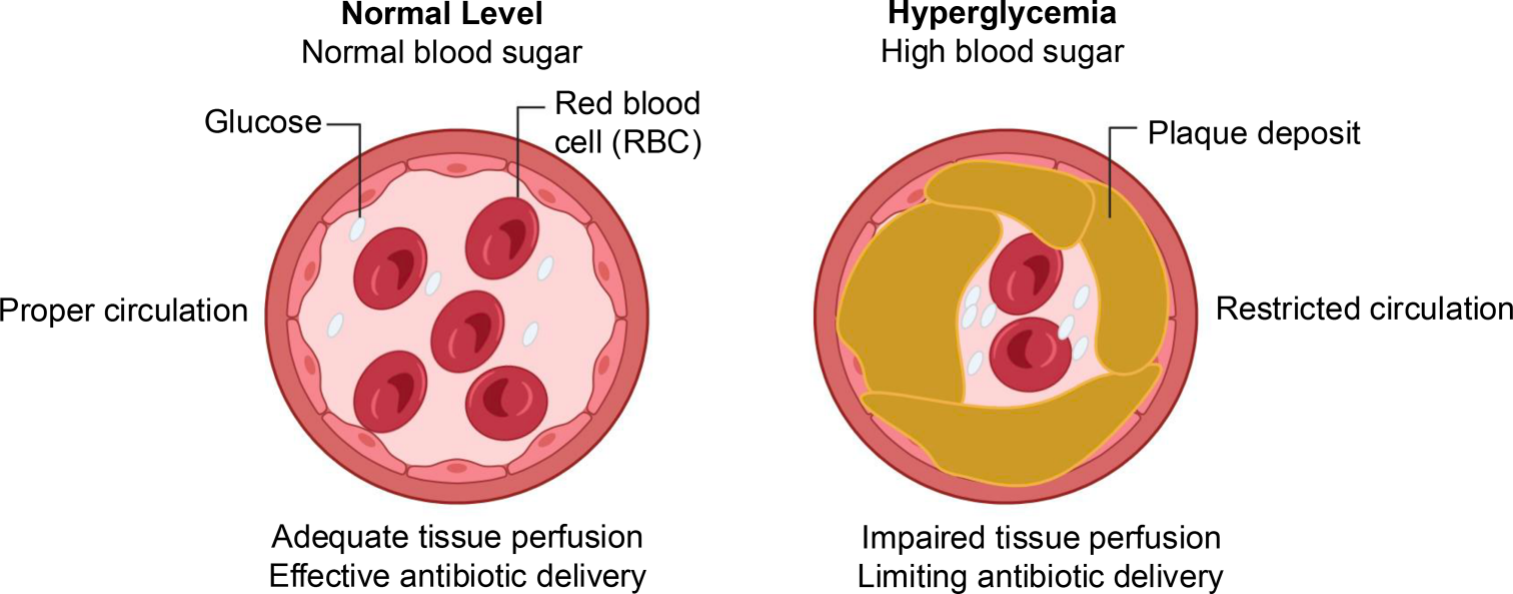
Vascular damage in diabetes limits antibiotic delivery. In normal physiological conditions, blood circulates efficiently through the vascular system, ensuring effective tissue perfusion and adequate delivery of nutrients, oxygen, and medicine (antibiotics) to the infected tissue (left). This warrants adequate therapeutic concentrations are achieved, allowing antibiotics to effectively eliminate the target bacteria. In individuals with hyperglycemia (high blood sugar), vascular plaque buildup restricts blood flow, limiting the delivery of nutrients, oxygen, and antibiotics to infected tissue (right). This leads to inadequate antibiotic delivery, resulting in subtherapeutic concentrations at the infected site. Consequently, infections persist, increasing the risk of treatment failure and the development of antibiotic resistance. Created in https://BioRender.com.

In individuals with diabetes and other metabolic diseases, vascular damage characterized by impaired circulation and capillary damage significantly compromises the effectiveness of antibiotic therapy (Rask-Madsen and King 2013, Omotosho et al. 2025). This vascular damage is largely driven by oxidative stress and chronic inflammation, which impair endothelium integrity, reduce NO bioavailability needed for vasodilation, increase arterial wall stiffness, and enhance atherosclerotic plaque buildup (Domingueti et al. 2016, Nedosugova et al. 2022, Li et al. 2023). Overtime, these changes narrow and block blood vessels, limiting tissue perfusion and thereby greatly obstructing the distribution of antibiotics to infected tissues (Fig. 4). This inadequate antibiotic delivery into tissues, especially in diabetic foot ulcers and ischemic limbs elevates the risk of treatment failure and foment the emergence of resistant bacterial strains (Baig et al. 2022, Fejfarová et al. 2024). Poor blood flow also creates low-oxygen environment that favor formation of bacterial biofilms, allowing infections to persist longer (Goswami et al. 2023, Fakher and Westenberg 2025). As a result, these individuals often require higher doses of antibiotics for longer duration. However, the impact of vascular impairment on antibiotic penetration varies across drug classes and depends on physio-chemical properties, such as the molecular size, lipophilicity, and protein-binding capacity. For example, because of vascular impairment, vancomycin shows reduced interstitial penetration in diabetic patients following cardiac surgery compared with nondiabetic controls (Skhirtladze et al. 2006). Conversely, ertapenem exhibits a different pattern, where total plasma concentrations are lower in diabetic patients receiving equivalent doses, but unbound interstitial tissue concentrations remain comparable to those of nondiabetic individuals. Maintaining active tissue levels of ertapenem results in better clinical outcomes in diabetic foot infections compared to other antibiotics (Sauermann et al. 2013). This demonstrates how vascular changes and drug properties together can limit antibiotic effectiveness in infected tissues of diabetic patients, requiring a more personalized approach to treat infections.

Renal dysfunction is another important factor that affects antibiotic treatment in individuals with metabolic disorders (Paudel et al. 2022, Abu-Humaidan et al. 2025). For example, diabetes can lead to kidney damage, called diabetic nephropathy, where high blood sugar damages the glomerular filtration process in the kidneys (Gross et al. 2005, Tonneijck et al. 2017). This reduces the kidneys’ ability to remove waste and excrete antibiotics, often prolonging the half-life of renally excreted drugs such as beta-lactams, aminoglycosides, glycopeptides, and fluoroquinolones (Whelton 1980, Aloy et al. 2020). The accumulation of some of these antibiotics can cause nephrotoxicity, leading to further kidney damage (Clifford et al. 2022, Campbell et al. 2023) (Fig. 3). Because of this toxicity, the antibiotics may need to be stopped, switched to another drug, or given at a lower dose, which can result in incomplete treatment. The bacteria exposed to low, sublethal dose of antibiotics undergo partial growth inhibition, which increases mutation accumulation and promotes antibiotic resistance (Gullberg et al. 2011, Khan et al. 2017, Wistrand-Yuen et al. 2018, Wang et al. 2019). Diabetic nephropathy also heightens the risk of infections by disrupting normal blader function and reducing urine flow, which normally helps flush bacteria from the bladder and urethra. In diabetic autonomic neuropathy, nerve damage impairs bladder control, causing incomplete bladder emptying and urine retention. This urinary stasis, along with glucose-rich urine creates an environment that favors bacterial growth, thereby increasing recurrent urinary tract infections and pyelonephritis (Hibbing et al. 2015, Agochukwu-Mmonu et al. 2020, Anandasekar et al. 2024, Vichayanrat et al. 2025). Repeated infections often require multiple courses or extended antibiotic therapy, which increases the likelihood of developing resistant bacteria (Shallcross et al. 2017, Carrillo-Larco et al. 2022). Therefore, tailoring antibiotic therapy for people with metabolic disease is crucial and demand personalized dosing and close therapeutic drug monitoring. This approach helps optimize treatment efficacy, minimize toxicity, and prevents bacteria from developing resistance overtime.

### Conclusions and perspectives

Frequent infections in individuals with metabolic disorders distinctly demonstrate the role of metabolism and the immune system in determining the host susceptibility to microbial invasion. The metabolic reprogramming of immune cells during infection and metabolic competition within infected tissues have been widely investigated (Mathis and Shoelson 2011, O’Neill et al. 2016, Horn and Kielian 2021), however, significant gaps remain, particularly their relevance in metabolic disorders. The situation is further complicated by the prevailing paradigm that considers infections to be primarily caused by planktonic bacteria, despite evidence that suggests that both acute and chronic infections are predominately associated with biofilm formation in the lungs and skin infection (Schaber et al. 2007, Kolpen et al. 2022a, 2022b). This highlights the importance of spanning the field of immunometabolism within biofilm biology to unlock novel strategies to overcome persistent and antibiotic-resistant infections.

Largely, interventions aimed at immune and metabolic pathways are highlighted as a potential adjunctive approach alongside antibiotics for managing infections (Helbig et al. 2013, Peng et al. 2025). The design of future studies that emphasize the early planktonic to biofilm transition in the microenvironment of metabolic disorders could disclose novel molecular targets that prevent onset of biofilms before tolerance mechanisms engage. With the rise of infections and metabolic disorders, and the expanding clinical use of GLP-1 receptor agonists (Yeo et al. 2024), further investigation is required to determine how their use influences immune cell plasticity and whether they affect pathogenic bacterial behavior. Although, GLP-1 receptor analogues have shown to promote changes in gut microbiome composition and microbial diversity, its effect on bacterial virulence pathways, EPS production and overall efficacy of antibiotics and other antimicrobials remains to be elucidated.

The transformation of spatial omics technologies has drastically improved our understanding of heterogenous ecosystem within the infected tissue across many bacterial infections (Seydel 2025). The scope of these innovative technologies can be extended to determine the spatial and temporal patterns of the metabolic and immune factors within the infected tissues of individuals with metabolic disease and people without these conditions. Future studies, by further integrating the spatial multiomics techniques for bacteria alongside host omics approaches, will provide a more comprehensive understanding of host–pathogen interactions.

Ultimately, the cooccurrence of metabolic and infectious diseases poses a significant challenge for public health. The combined impact of these diseases places a substantial economic burden, including direct costs associated with diagnosis, medication, and hospitalization and in indirect costs related to loss of productivity. It is long overdue to accept the coexistence of metabolic diseases and chronic infections as a cohesive clinical problem and shift from reactive treatment to integrated prevention. Furthermore, nurturing cross-disciplinary collaborations across immunology, microbiology, endocrinology, and computational biology, together with new and emerging artificial tools is essential for the overall benefit of individuals living with these conditions.
